# Metastatic Malignant Ectomesenchymoma Initially Presenting as a Pelvic Mass: Report of a Case and Review of Literature

**DOI:** 10.1155/2014/792925

**Published:** 2014-10-27

**Authors:** A. Nael, P. Siaghani, W. W. Wu, K. Nael, Lisa Shane, S. G. Romansky

**Affiliations:** ^1^Department of Pathology and Laboratory Medicine, University of California Irvine Medical Center, 101 The City Drive, Orange, CA 92868, USA; ^2^Department of Pathology and Laboratory Medicine, Memorial Sloan Kettering Cancer Center, 1275 York Avenue, New York, NY 10065, USA; ^3^Department of Medical Imaging, University of Arizona Medical Center, 1501 N. Campbell Avenue, P.O. Box 245067, Tucson, AZ 85724-5067, USA; ^4^University of California Irvine, Long Beach Memorial Care Health System, 2801 Atlantic Avenue, Long Beach, CA 908068, USA

## Abstract

Pediatric soft tissue sarcomas account for approximately 10% of all pediatric malignancies. Malignant ectomesenchymoma is rare biphasic sarcomas consisting of both mesenchymal and neuroectodermal elements. Approximately 64 cases have been reported in the literature and are believed to arise from pluripotent embryologic migratory neural crest cells. We report a 4-year-old boy who initially presented with a pelvic mass and inguinal lymphadenopathy at 6 months of age. Inguinal lymph node biopsy revealed a distinct biphasic tumor with microscopic and immunophenotypic characteristics diagnostic for both alveolar rhabdomyosarcoma and poorly differentiated neuroblastoma. The patient received national protocol chemotherapy against rhabdomyosarcoma with good response and presented with a cerebellar mass 21 months later. The metastatic tumor revealed sheets of primitive tumor cells and diagnostic areas of rhabdomyosarcoma and neuroblastoma were identified only by immunohistochemistry. Cytogenetic analysis of metastatic tumor demonstrated complex karyotype with multiple chromosomal deletions and duplications. The patient received national protocol chemotherapy against neuroblastoma and adjuvant radiotherapy after surgical resection of the cerebellar tumor with good response. He is currently off from any treatment for 18 months with no evidence of tumor recurrence or metastasis.

## 1. Introduction

Pediatric soft tissue sarcomas account for approximately 10% of all pediatric malignancies and are considered the fifth most common pediatric soft tissue neoplasm following leukemia/lymphoma, central nervous system tumor, neuroblastoma, and Wilms' tumor [1]. Malignant ectomesenchymoma (MEM) is a rare soft tissue sarcoma with a biphasic morphology consisting of both mesenchymal and neuroectodermal elements such as rhabdomyosarcoma (RMS) and ganglioneuroblastoma. MEMs are believed to arise from pluripotent embryologic migratory neural crest cells able to form both mesenchymal and neuroectodermal tissues [2]. Because these cells are widely distributed throughout the body, MEMs may arise in diverse sites but the most common reported location is perineal/pelvic area [3]. These tumors are exceedingly rare and approximately 64 cases have been reported in English literature in all age groups with preponderance in the first decade of life [3]. Due to the rare incidence of MEM, our knowledge of tumor genetics, biological behavior, treatment, outcome, and prognosis is limited.

## 2. Case Report

**Figure 1:**
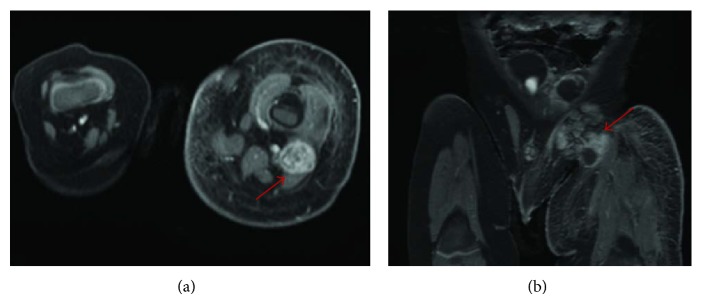
Axial (a) and coronal (b) T1-contrast-enahnced MR images through the thighs are shown. There is a heterogeneously enhancing mass in the posterior thigh involving the adductor compartment (arrow in (a)). There are also several enlarged external iliac lymph nodes: some with cystic and necrotic changes (arrow in (b)). Note the enlargement of the left lower extremity and significant soft-tissue edema and fat stranding.

**Figure 2:**
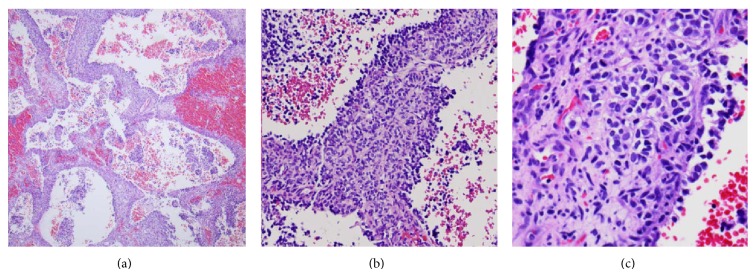
Histologic features of tumor in the left inguinal lymph node. (a) The RMS-like component showed variably sized cystic spaces separated by fibrovascular septa. (b) Cystic spaces lined by loosely cohesive primitive cells floating into spaces, imparting an alveolar pattern. (c) The tumor cells demonstrated nesting pattern within the fibrovascular septa (hematoxylin-eosin, original magnification ×40 (a); original magnification × 200 (b); original magnification ×400 (c)).

**Figure 3:**
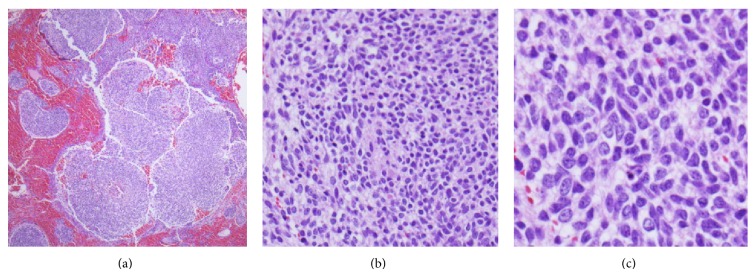
Histologic features of tumor in the left inguinal lymph node. (a) Neuroblastoma component with nodular growth pattern. (b) Each nodule is composed of primitive neuroblasts with scant amount of neuropil. (c) Neuroblasts with salt and peppery nuclei and low MKI (hematoxylin-eosin, original magnification ×40 (a); original magnification ×200 (b); original magnification ×400 (c)).

**Figure 4:**
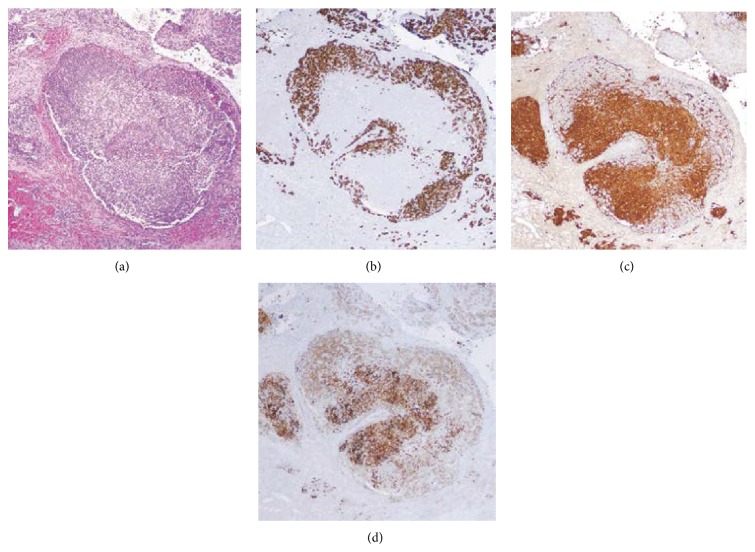
Immunohistochemical features of tumor in the left inguinal lymph node. (a) Microscopic photographs from left inguinal lymph node biopsy reveal primitive tumor cells with nodular growth pattern. The tumor cells demonstrate immunohistochemical reactivity for (b) myogenin, (c) PGP9.5, and (d) tyrosine-hydroxylase to show both myogenic and neural differentiation (original magnification ×200 (a–d)).

**Figure 5:**
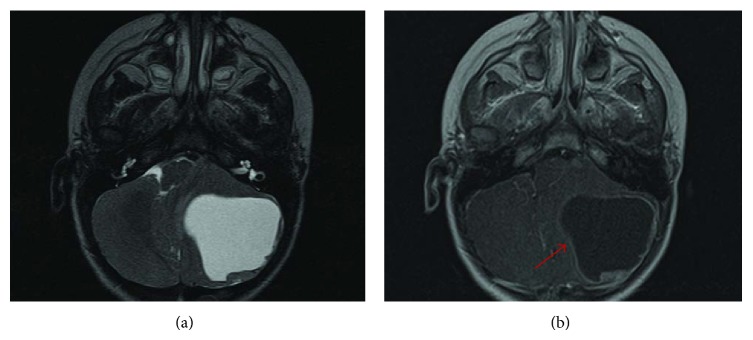
Axial T2 (a) and T1-contrast-enhanced (b) MR images of brain. There is a 5.6 × 5.1 cm largely cystic mass with peripheral nodular enhancement (arrow in (b)) involving the left cerebellar hemisphere. There is mass effect with compression of the 4th ventricle and effacement of the left premedullary cistern.

**Figure 6:**
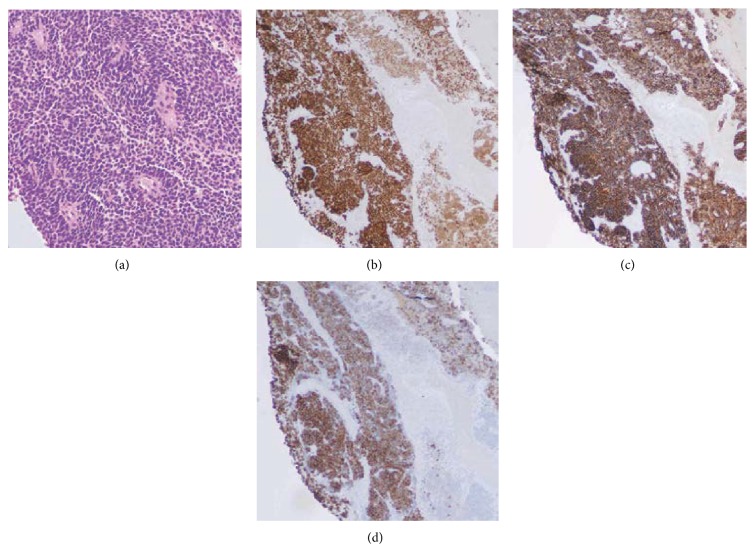
Histologic and immunohistochemical features of tumor in the left cerebellum. (a) Microscopic photographs from left cerebellar resection show sheets of primitive tumor cells with neuroblastic rosettes resembling a primary medulloblastoma. The tumor cells demonstrate immunohistochemical reactivity for (b) myogenin, (c) CD56, and (d) tyrosine-hydroxylase to show both myogenic and neural differentiation (original magnification ×200 (a–d)).

Our patient is a 4-year-old Hispanic boy. He first presented at 6 months old to the Emergency Room with a chief complaint of left leg swelling and pain for a month. Further work-up including pelvic and thigh magnetic resonance imaging (MRI) revealed a heterogeneous partially cystic enhancing bilobed mass at the left side of the pelvis, measuring 5.7 × 4.3 × 4.0 cm (Figure 1). The left external iliac artery and vein coursed between the two lobes of the mass. In addition, multiple enlarged left inguinal lymph nodes were identified with solid and cystic appearance, suggestive of tumor metastasis. Diagnostic excisional inguinal lymph node biopsy was done. Sections revealed a distinct biphasic appearance by light microscopy (Figures 2 and 3) and immunohistochemical analysis (Figure 4) demonstrated both alveolar rhabdomyosarcoma-like (ARMS-like) and poorly differentiated neuroblastoma components. No evidence of residual lymph node was identified. The RMS component was composed of prominent spaces separated by fibrovascular septa (Figure 2(a)). The septa were lined by loosely cohesive primitive cells with hyperchromatic nuclei and variable amount of scant cytoplasm, imparting an alveolar pattern (Figure 2(b)). However, there were foci where tumor cells demonstrated nesting pattern within the fibrovascular septa with pleomorphic nuclei (Figure 2(c)). The neuroblastoma component showed schwannian stroma poor tumor with more primitive neuroblasts and scant amount of neuropil in a nodular growth pattern (Figures 3(a) and 3(b)). Moreover, the neuroblastic tumor cells showed speckled salt and pepper nuclei, inconspicuous nucleoli, and little nuclear pleomorphism with a variable amount of scant cytoplasm. The mitotic-karyorrhectic index (MKI) was low (<2%) (Figure 3(c)). The RMS component was strongly positive for myogenin (Figure 4(b)) and desmin by immunohistochemical staining, while the neuroblastoma component was stained with neural markers such as PGP9.5 and tyrosine-hydroxylase (Figures 4(c) and 4(d)), CD56, synaptophysin, and S100. Whole body work-up including MRI, positron emission tomography scan (PET scan), and bone marrow biopsy did not show any evidence of tumor involvement in other areas of the body including the central nervous system. Due to the extensive lymphadenopathy in the pelvic and inguinal area, the patient's tumor was considered to be metastatic and treated against RMS as it was the more aggressive component of the tumor. He received and completed national protocol chemotherapy for ARMS (COG-ARST08P1 protocol [22]), with significant reduction in his tumor burden. He was doing well and had been off of chemotherapy for about four months, when he became less active and showed ataxic gait with episodes of vomiting, 21 months after first presentation. MRI of the brain showed a 5.6 × 5.1 × 4.2 cm left cerebellar cystic mass with thick peripheral enhancement and some hemorrhage, consistent with metastasis (Figure 5). The tumor showed significant mass effect on the fourth ventricle and brain stem. There was no evidence of tumor recurrence or metastasis in other sites. Due to the location of the tumor, mass effect, and tumor size reduction, excisional surgery was done. The metastatic tumor displayed a more homogenous microscopic appearance with sheets of primitive tumor cells resembling a primary medulloblastoma (Figure 6(a)). Diagnostic areas of RMS and neuroblastoma were only identified by immunohistochemistry demonstrating strong positivity for myogenin, CD56, and tyrosine hydroxylase (Figures 6(b)–6(d)). However, neuroblastoma was the predominant component. Peripheral blood chromosome analysis revealed a normal male chromosome complement (46, XY) with no abnormalities. Molecular analysis utilizing reverse transcription polymerase chain reaction (RT-PCR) was performed on the cerebellar tumor and showed no evidence of a PAX3-FOXO1 t(2;13) (q35;q14) or a PAX7-FOXO1 t(1;13) (p36;q14) chromosomal translocation. Although fluorescence in situ hybridization (FISH) studies revealed FOXO1 (FKHR) gain on chromosome 13q14.11 in 75% of the tumor cells, there was no PAX-FOXO1 translocation. Tumor chromosome analysis showed complex karyotype with near-triploid cell line (71, XYY) and multiple chromosomal deletions (chromosomes 3 and 4) and duplications (chromosomes 5, 7, 19, and 22). After surgical resection, he received national chemotherapy protocol against the neuroblastoma component (COG-ANBL0532 protocol [23]), which was the most prominent component of the metastatic tumor. In addition, he received adjuvant local radiation therapy. He completed his chemoradiation therapy with excellent tumor response. Currently he is not receiving any additional treatments for about 18 months and his most recent follow-up MRI and PET scan did not show any evidence of residual or metastatic tumor. We report another MEM case with cytogenetic analysis, as there are only 5 reported cases in the literature with these data. Moreover our case emphasizes the importance of multimodality treatment approach in prognosis, even in nonresectable primary tumors.

## 3. Discussion

**Figure 7:**
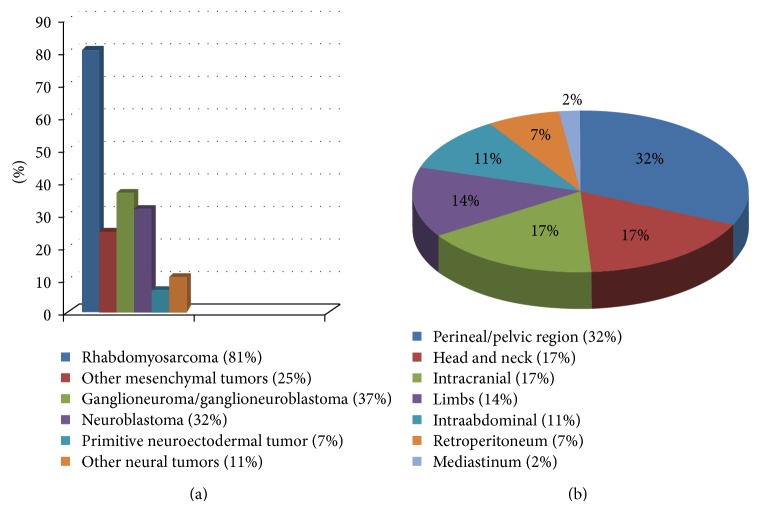
Charts to show (a) histological features and (b) primary anatomical sites of involvement of malignant ectomesenchymoma.

**Figure 8:**
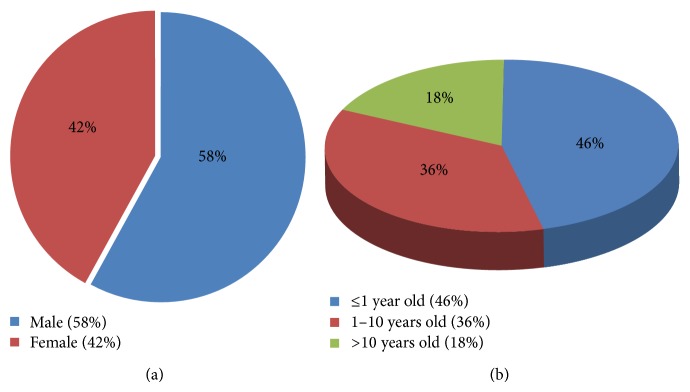
Charts to show (a) incidence according to sex and (b) incidence according to age of malignant ectomesenchymoma.

Across all ages with MEM, the mesenchymal component is generally RMS with predominantly embryonal subtype [2, 3, 7, 24–27] but pleomorphic sarcoma, undifferentiated sarcoma, chondrosarcoma, liposarcoma, and gliosarcoma have been reported [3, 11, 21]. The neuroectodermal component can be highly variable ranging from clustered ganglion cells to immature primitive neural elements only identified by immunohistochemical staining [2, 11, 24–27]. Freitas et al. have reported 40 MEM cases from 1946 to 1998 with related data regarding the sex, age, primary site, histology pattern, treatment, and survival from the time of presentation. After reviewing the English literature from 1998 to the present, we found additional 24 MEM cases, which have both microscopic and immunophenotype characteristics of MEM (Table 1). Combining data from the Freitas et al. study and our observation revealed RMS and ganglioneuroma/ganglioneuroblastoma with clustered or scattered ganglion cells are the most common histological patterns seen in MEM cases (Figure 7(a)). Moreover, the most common site of presentation is the perineal/pelvic area, followed by head and neck, intracranial, limbs, intra-abdominal, and retroperitoneal (Figure 7(b)). While some reports support the idea of MEM having male predilection and occurring typically in infancy [2, 7, 24, 26], other studies do not show this predilection [3, 13, 27]. Our observation shows these tumors to have a slightly male predominance (male to female ratio of 1.4) and most commonly present in the first decade of life (82%) (Figure 8). Our case showed RMS as mesenchymal component but with alveolar pattern and poorly differentiated neuroblastoma as neuroectodermal component. The area resembling RMS has both histological and immunohistochemical staining pattern typical of alveolar type RMS. FISH analysis failed to detect any of the two recurrent chromosomal translocations commonly seen in alveolar rhabdomyosarcoma (ARMS) such as t(2;13)(q35;q14), seen in 55% of the cases, or t(1;13)(p36;q14), seen in 22% of cases [28, 29]. In addition to our case, there are five reports of MEM in the literature with cytogenetic analysis. Karyotyping analysis of malignant ectomesenchymoma cases is shown as follows.

*Case  1*. A 5-month-old girl with pelvic mass [25]:

 49,XY, +8, +8, +11/49,XY, +2, +11, +11/46,XX.

*Case  2*. A 16-month-old boy with abdominal mass [30]:

 53,XY, +2, add(6)(p24), +8, +8, +9, +10, +11, t(12;15)(p12;q24), +20.

*Case  3*. An 8-month-old boy with scrotal mass [15]:

 49,XY, +2, −6, +11, +20, +mar(chromosome 6 material by florescent in situ hybridization).

*Case  4*. A 4-year-old girl with intracranial mass [14]:

 84–87, XXX, −X, −1, der(2)t(1;2)(q12;q14.1), −4, −5, −5,
 der(5)t(5;?;5)(p15;?;q13)x2, −9, −9, del(11)(q22)x2, −17, −19, −21,
 der(21)t(17;21)(q21;q22), −22, −22, +r, +mar1, +mar2, mar3[cp10].

*Case  5*. A 6-month-old girl with protruding vaginal mass [18]:

 46,XX,der(1)t(1;12)(p32;p13)inv(1)(p13q25),del(5)(q13q22),
 der(12)t(1;12)(p32;p13)[9]/46,XX [3].

*Case  6*. A 6-month-old boy with pelvic mass (our case):

 71, XYY, add(1)(p13), −3, −4, +5, +7, +19, +22.

Four of these cases had complex karyotypes. Trisomies 2, 8, and 11 were the most commonly reported genetic abnormalities [14, 15, 25, 30]. One case demonstrated a t(1;12) translocation without ETV6 rearrangement as seen in congenital cellular mesoblastic nephroma [18]. In our case the tumor chromosome analysis revealed a complex karyotype with near-triploid cell line and multiple chromosomal deletions and duplications (71〈3n〉, XYY, add (1) (p13), −3, −4, +5, +7, +19, +22), none of which were tumor specific (Table 1). Since MEM is a biphasic tumor with variable differentiation and percentage of its components, it can be in the differential diagnosis of well differentiated to poorly differentiated mesenchymal sarcomas or neuroectodermal tumors such as embryonal rhabdomyosarcoma (ERMS), ARMS, pleomorphic sarcoma, chondrosarcoma, undifferentiated sarcoma, ganglioneuroma, neuroblastoma, peripheral primitive neuroectodermal tumor (pPNET), and malignant schwannoma [2, 21]. However, to diagnose MEM, there must be both mesenchymal and neural elements with immunohistochemical reactivity for myogenin and/or desmin, CD56, PGP9.5, synaptophysin, chromogranin, and tyrosine hydroxylase [2, 27]. Due to the rarity of MEM, data regarding treatment and prognosis is limited. Most investigators suggest a multimodality treatment approach including surgery, chemotherapy, and radiation therapy as these tumors almost will act and have the same prognosis as RMS-like soft tissue sarcomas [2, 31]. In fact, when the predominant mesenchymal element in MEM is RMS, the overall outcome and prognosis are similar to RMS; thus, underdiagnoses may not have a major impact on clinical treatment [27]. In such cases, the International Rhabdomyosarcoma Study Group-IV (IRS-IV) recommends that risk stratification and treatment planning should be done based on age, pretreatment stage (including tumor size, tumor site, regional lymph node status, and disseminated disease), and postoperative clinical grouping depending on completeness of disease resection and lymph node status [2, 7, 27, 32]. Based on this study, for localized disease surgical resection with clear margins and additional chemotherapy is favored [32]. However, for disseminated disease chemotherapy is preferred and tumor debulking is not recommended. Instead, a biopsy should be provided to confirm the diagnosis [33]. Moreover, consideration of additional radiation therapy depends on postoperative clinical grouping. Some studies have demonstrated that the most important independent prognostic factor in MEM cases is tumor resectability as most patients who have died of disease had an unresectable primary tumor or metastasis at the time of presentation [11, 12]. Similar to chemotherapy for other biphasic tumors, in cases where chemotherapy is the mainstay option, agents targeting the most aggressive component are chosen, which is RMS in MEM cases [2]. However, initial reports have shown MEM to have a poor prognosis [7, 24]. Case reviews [34] from 2005 and 2013 revealed MEM to have the same prognosis as other pediatric chemotherapy-sensitive soft tissue sarcomas, with 71% (15/21) and 83% (5/6) of children with MEM surviving following multimodality treatment approach, respectively [2, 11]. Finally, as these tumors have different morphology and genetics from other soft tissue sarcomas, further investigation is necessary to better understand the tumor biology and behavior with the hope of improving treatment protocols and ultimately patient prognosis.

## Figures and Tables

**Table 1:** Review of malignant ectomesenchymoma cases reported after 1998.

Case number	Age^a^	Sex	Primary site	Histology^b^	Recurrence or metastasis^a^	Treatment^c^	Follow-up^a^
1 [4]	13 yr.	M	Scrotum	ERMS and GCs	Retroperitoneum Met. after 2 yr.	DS, CT, and RT	NA

2 [5]	10 yr.	F	Intracranial	US with rhabdoid features and NB	Local Rec. after 5 wk.	TSR	NED after 12 mo.

3 [6]	19 mo.	M	Pelvic	ERMS and NB	Local Rec. and BM Met. after 8 yr.	TSR and CT	NED for 8 yr., NA after Met.

4 [7]	11 mo.	M	Intra-abdomen	US with rhabdoid features and NB	Liver, lung, and BM Met. at the time of presentation	DS, CT, and RT	DOD after 9 mo.

5 [8]	61 yr.	M	Retroperitoneum with invasion to vertebral bone	ERMS and GN	No	DS and RT	DOD after 14 mo.

6 [9]	1.5 yr.	M	Upper lip	ERMS and GCs	Local Rec. after 1 yr.	TSR and CT	NED for 1 year, NA after Rec.

7 [10]	4 mo.	M	Pelvic	ERMS, GCs, and schwannoma	NA	TSR and CT	NA

8 [11]	17 mo.	M	Left wrist	RMS, CRS, GNB	No	TSR and CT	NED after 4 yr.

9 [12]	10 yr.	F	Intracranial	ERMS and NB	No	TSR, CT, and RT	NED after 6 yr.

10 [13]	10 d.	F	Face	RMS and GCs	No	Biopsy and CT	DOD, after a few days

11 [14]	4 yr.	F	Intracranial	US with focal rhabdomyoblastic diff. and GCs	Lung Met. at the time of presentation	TSR and CT	DOD after 10 wk.

12 [15]	8 mo.	M	Scrotum	ERMS and GC	NA	TSR and CT	NA

13 [16]	10 yr.	M	Intracranial	US and GCs	No	TSR, CT, and RT	NED after 20 mo.

14 [17]	36 yr.	F	Ethmoid sinus and orbit	RMS and NB	No	Biopsy, CT, and RT	NED after 28 mo.

15 [18]	6 mo.	F	Vagina	ERMS and GCs	Abdomen-pelvic Met. after 4 mo.	DS and CT	DOD after 15 mo.

16 [19]	43 yr.	F	Nasal cavity	RMB and NB	No	Biopsy, CT, and RT	NED after 10 mo.

17 [20]	6 yr.	M	Intracranial, frontal lobe	US and GCs	No	TSR, CT, and RT	NED after 2 years

18 [2]	4 yr.	F	Orbit	ERMS and NB	No	TSR, CT, and RT	NED after 12.9 years

19 [2]	2.5 mo.	F	Upper arm	ARMS and pPNET	No	TSR and CT	NED after 13.7 years

20 [2]	13.5 yr.	M	Buttock	ARMS and NB	Local Rec. and lungs Met. after 1.1 yr.	DS, CT, and RT	DOD after 1.3 years

21 [2]	1 yr.	M	Groin	ERMS and NB	No	TSR and CT	NED after 5 years

22 [2]	7 mo.	F	Sole	ERMS and NB	Local Rec. after 5 mo.	TSR and CT	NED after 2.3 years

23 [2]	8 mo.	M	Intra-abdomen	ERMS and NB	Local Rec. after 1.4 yr.	TSR and CT	NED after 2.1 years

24 [21]	5 mo.	M	Mediastinum with invasion into lung and SVC	RMS and pPNET	No	DS and CT	DOD after 11 mo.

25 (Our case)	6 mo.	M	Inguinal and pelvic	ARMS and NB	Cerebellum Met. after 21 mo.	Biopsy, CT, and RT	NED after 3 yr.

ARMS, alveolar rhabdomyosarcoma; BM, bone marrow; CRS, chondrosarcoma; CT, chemotherapy; diff., differentiation; DOD, dead due to disease; DS, debulking surgery; ERMS, embryonal rhabdomyosarcoma; F, female; GC, ganglion cell; GN, ganglioneuroma; GNB, ganglioneuroblastoma; M, male; Met., metastasis; mo., month(s); NA, no data available; NB, neuroblastoma; NED, no evidence of disease; pPNET, peripheral primitive neuroectodermal tumor; Rec., recurrence; RMB, rhabdomyoblastoma; RMS, rhabdomyosarcoma; RT, radiation therapy; SVC, superior vena cava; TSR, total surgical resection; US, undifferentiated sarcoma; wk, week(s); yr., year(s); ^a^age, recurrence/metastasis and follow-up since first diagnosis; ^b^itdescribes which tumor components were present in respect to diagnosis of MEM; ^c^itdescribes type of treatment on the primary tumor.
